# Systematic review of photobiomodulation for multiple sclerosis

**DOI:** 10.3389/fneur.2024.1465621

**Published:** 2024-09-12

**Authors:** Vander Oliveira de Andrade Filho, Marina Oliveira Coura Amarante, Francisco Gonzalez-Lima, Sérgio Gomes da Silva, Fabrízio dos Santos Cardoso

**Affiliations:** ^1^Centro Universitário FAMINAS, Muriaé, MG, Brazil; ^2^Department of Psychology and Institute for Neuroscience, University of Texas at Austin, Austin, TX, United States; ^3^Hospital do Câncer de Muriaé, Fundação Cristiano Varella (FCV), Muriaé, MG, Brazil; ^4^Centro Universitário Redentor (UniREDENTOR/Afya), Itaperuna, RJ, Brazil

**Keywords:** photobiomodulation, low-level laser therapy, multiple sclerosis, autoimmune encephalomyelitis, demyelination

## Abstract

**Background:**

Multiple sclerosis (MS) is an inflammatory chronic autoimmune and neurodegenerative disorder of the brain and spinal cord, resulting in loss of motor, sensorial, and cognitive function. Among the non-pharmacological interventions for several brain conditions, photobiomodulation (PBM) has gained attention in medical society for its neuroprotective effects. We systematically reviewed the effects of PBM on MS.

**Methods:**

We conducted a systematic search on the bibliographic databases (PubMed and ScienceDirect) with the keywords based on MeSH terms: PBM, low-level laser therapy, multiple sclerosis, autoimmune encephalomyelitis, demyelination, and progressive multiple sclerosis. Data search was limited from 2012 to July 2024. We followed the Preferred Reporting Items for Systematic Reviews and Meta-Analyses (PRISMA) guidelines. The initial systematic search identified 126 articles. Of these, 68 articles were removed by duplicity and 50 by screening. Thus, 8 studies satisfied the inclusion criteria.

**Results:**

The reviewed studies showed that PBM modulates brain markers linked to inflammation, oxidative stress, and apoptosis. Improvements in motor, sensorial, and cognitive functions in MS patients were also observed after PBM therapy. No study reported adverse effects of PBM.

**Conclusion:**

These findings suggest the potential of PBM as a promising non-pharmacological intervention for the management of MS, although further research is needed to standardize PBM protocols and assess its long-term effects.

## 1 Introduction

Multiple sclerosis (MS) is an inflammatory, chronic autoimmune, and neurodegenerative disorder of the brain and spinal cord that results in loss of motor, sensorial, and cognitive function (1–3). According to the National Multiple Sclerosis Society, MS affects more than 2 million people worldwide (3, 4).

This disorder starts with an inflammatory cascade in the central nervous system (CNS), which is caused by inappropriately activated T cells which in turn induces an immune response against myelin and oligodendrocytes (1, 5–7).

Clinically, MS begins with discrete episodes of neurological dysfunction followed by partial, complete, or no remission. Over time, most patients develop a sustained accumulation of disability, known as secondary progressive MS (SPMS) (8, 9). About 10% of MS develop accumulation of disability from clinical onset with no reporting a preceding period of clinical relapses and remissions and are known as primary progressive MS (PPMS) (9). Despite these different classifications, all clinical forms of MS seem to reflect the same underlying disease process (10).

Photobiomodulation (PBM) is a non-invasive technique that uses red-to-near-infrared light to stimulate wound healing, reducing pain and inflammation in several diseases (11). PBM also improves brain functions in several conditions (12–15). For example, Disner et al. (14) reported that transcranial PBM at 1064 nm wavelength reduces depression symptoms in participants with better response to attention bias modification. Vargas et al. (15) observed that infrared PBM at 1064 nm, 250 mW/cm^2^, improved the cognitive function and EEG rhythms of older adults with memory complaints. Animal studies have observed similar effects (16–18). Salehpour et al. (18) reported that PBM prevented cognitive impairment induced by sleep-deprived. In addition, PBM enhanced the antioxidant status and increased mitochondrial activity in the hippocampus of sleep-deprived mice. Our research group noted that PBM increased the levels of interleukin-1α (IL-1α) and decreased the levels of IL-5 and the expression of p38 stress-activated protein kinase (p38) in both the cortex and hippocampus of aged rats (16). These promising effects of PBM have been investigated from various perspectives, including in neuroinflammatory response (19, 20).

Based on the well-documented therapeutic effects of PBM in different neurological conditions (16–18, 21–24), we systematically review the effects of PBM in MS.

## 2 Methods

### 2.1 Search strategy and sources

We used the PubMed and ScienceDirect databases with the keywords based on MeSH terms: photobiomodulation, low-level laser therapy, multiple sclerosis, autoimmune encephalomyelitis, demyelination, and progressive multiple sclerosis. Data search was limited from 2012 to June 2024. This study followed the guideline of Preferred Reporting Items for Systematic Reviews and Meta-Analyses (PRISMA) (25). Two evaluators made the evaluations, and disagreements were resolved by consensus.

### 2.2 Selection criteria

The search strategy included experimental and clinical studies using PBM in MS. We included original *in vitro* and clinical articles written in English.

### 2.3 Data extraction and data synthesis

For data extraction, we used an individualized data form (26), in which articles were subdivided according to the author, subjects, light source, PBM parameters (center wavelength, operation mode, average radiant power, irradiance at aperture, beam spot size, exposure duration, radiant exposure, number of points irradiated, number of sessions, total radiant energy) and outcomes. The data are presented in the Results section.

## 3 Results

### 3.1 Study selection

**Figure 1:**
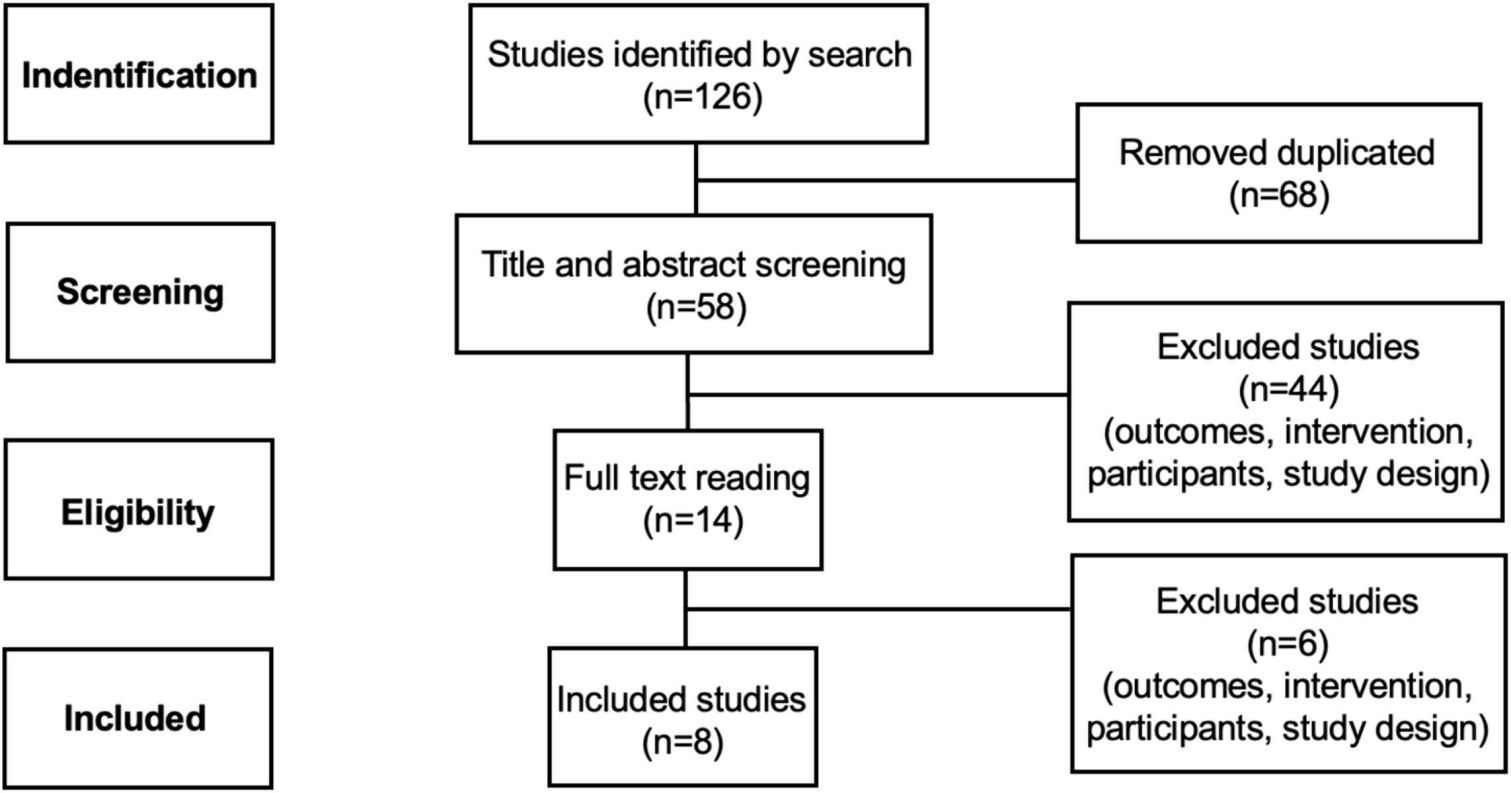
Summary of article search and selection process.

The initial database search resulted in 126 studies. Of these, 68 articles were removed due to duplicity, 50 were screened out, and 8 studies were included in the systematic review. The process of selecting the articles is illustrated in Figure 1.

### 3.2 Study characteristics

Four articles reported experiments in mice (27–30), of which 2 were on female C57BL/6 (29, 30) and 2 were on male C57BL/6 (27, 28) (Table 1). In these studies, the age of animals varied from 6 to 10 weeks old. In humans, 4 articles reported a randomized clinical trial in men and women aged 18–60 diagnosed with MS (31–34) (Table 2). In all studies, the subjects received PBM treatment. The studies aimed to analyze the effects of PBM on the damages caused by MS, inflammatory response and oxidative stress, mitochondrial activity, demyelination, microglial modulation, and apoptosis.

**Table 1:** Evidence of PBM in animal models of MS.

Author	Subjects	Light source	PBM parameters	Outcomes
(29)	Female C57BL/6	LED	Center wavelength: 670 nm Average radiant power: 2100 mW Exposure duration: 180 s Radiant exposure: 5 J/cm^2^ Number of sessions: 7 Total radiant energy: 375J	PBM reduced mean clinical scores. In addition, PBM decreased IFN-γ and TNF-α levels, and increased IL-4 and IL-10 levels.
(30)	Female C57BL/6	LED	Center wavelength: 670 nm Average radiant power: 2100 mW Exposure duration: 180 s Radiant exposure: 5J/cm^2^ Number of sessions: 7 Total radiant energy: 375J	PBM attenuated antigen-specific nitric oxide. Also, PBM exhibited up-regulation of the Bcl-2 anti-apoptosis gene, and increased Bcl-2: Bax ratio.
(28)	Male C57BL/6 mice (6–10 weeks of age)	LED	Center wavelength: 660 and 904 nm Operation mode: continuous and pulsed Average radiant power: 30 mW and 70 W, pulsed regime (time of pulse 60 ns) Beam spot size: 0.06 and 0.10 cm^2^ Exposure duration: 20 s for each position Radiant exposure: 10 and 3J/cm^2^ Number of points irradiated: 6 Number of sessions: 30 Total radiant energy: 0.6J	PBM inhibited clinical signs, neuroinflammation, and oxidative damage induced by encephalitogenic T lymphocytes and microglia in the brain.
(27)	Male C57BL/6 mice (7 weeks of age)	LED	Center wavelength: 808 nm Operation mode: continuous Average radiant power: 50 mW Irradiance at aperture: 1.78 W/cm^2^ Beam spot size: 0.028cm^2^ Exposure duration: 20s Radiant exposure: 36J/cm^2^ Number of points irradiated: 1 Number of sessions: 6 Total radiant energy: 1J	PBM increased motor performance, attenuated demyelination, increased the number of oligodendrocyte precursor cells, modulated microglial and astrocyte activation, and milder toxicity by cuprizone.

Photobiomodulation (PBM); interferon gamma (IFN-γ); Tumor necrosis factor-alpha (TNF-α); Interleukin-4 (IL-4); Interleukin-10 (IL-10); B cell lymphoma-2 (Bcl-2); BCL-2-associated X (BAX).

**Table 2:** Evidence of PBM in MS patients.

Author	Subjects	Light source	PBM parameters	Outcomes
(33)	MS patients	LASER	Center wavelength: 830 nm Average radiant power: 15 mW Irradiance at aperture: 0.17 W/cm^2^ Exposure duration: 2400 s Number of points irradiated: 4 Number of sessions: 24	PBM reduces pain and improves range of motion
(31)	Individuals with a diagnosis of MS (EDSS)	LASER	Center wavelength: 650 nm Average radiant power: 50 mW Beam spot size: 1 cm^2^ Exposure duration: 30 s Number of points irradiated: 20 Number of sessions: 21 Total radiant energy: 3J	PBM improves the functional status of patients.
(34)	Individuals with a diagnosis of MS (EDSS)	LED	Center wavelength: 808 nm Operation mode: continuous Average radiant power: 100 mW Irradiance at aperture: 0.80 W/cm^2^ Beam spot size: 0.13 cm^2^ Exposure duration: 360 s Radiant exposure: 287J/cm^2^ Number of sessions: 24 Total radiant energy: 36.5J	PBM increased the expression of IL-10.
(32)	MS patients	LASER/LED	Center wavelength: 640 nm, 875 nm, and 905 nm Operation mode: pulsed laser Irradiance at aperture: 0.033 W/cm^2^ Exposure duration: 300 s, 600 s, and 900 s Number of sessions: 4 Total radiant energy: 40J, 80J, and 120J	PBM improved force recovery and muscle strength.

Photobiomodulation (PBM); Interleukin-10 (IL-10).

## 4 Discussion

This systematic review aimed to investigate the effects of PBM therapy in MS. Studies have shown that laser therapy reduces the clinical signs of disease and demyelination and exhibits anti-inflammatory and antioxidant properties. In addition, PBM increases the expression of genes linked to cellular proliferation and reduces apoptosis.

### 4.1 PBM-induced changes in clinical signs

Studies show that the laser improves clinical signs in patients with MS (31–33). In the study conducted by Seada et al. (33), the laser treatment performed three times per week on consecutive days (24 sessions) reduced trigeminal pain and increased mouth opening, masseter, and temporalis muscles. In another study, Kubsik et al. (31) observed that, after PBM treatment with 21 sessions, MS patients improved their functional status based on Expanded Disability Status Scale (EDSS) of Kurtzke and Barthel Index. Also, Rouhani et al. (32) noted an improvement in force recovery of patients treated with 4 sessions of PBM treatment. These therapeutic effects of PBM have also been observed in laboratory animals (27–29). Among the models studied in the literature are: experimental autoimmune encephalomyelitis (EAE) and cuprizone intoxication (35–38). In these models, the onset and progression of the disease are determined by a clinical score based on the progressive appearance of paralysis, the origin of locomotor deficits, and the gradual disability of the pathology (35, 36). In the study conducted by Gonçalves, thirty sessions of PBM (660 nm and 904 nm) were able to reduce clinical score and delay the disease onset in EAE mice. Also, a body weight gain was observed in the animals from the laser group. In the study by Duarte (27), cuprizone-induced MS model animals that received six sessions of laser treatment (808 nm) applied on three consecutive days for 2 weeks showed an improvement in motor performance. These data suggest that PBM improves the clinical signs of MS.

### 4.2 Anti-inflammatory effects of PBM

The diagnosis of MS is established in conjunction with the clinical presentation and inflammatory lesions (39). In this sense, studies performed on patients (34) and animal models of MS (27, 28, 30) show that PBM modulates the levels of inflammatory markers. For example, after 24 sessions of PBM twice a week, the expression of IL-10 increased significantly in MS patients (34). It is known that the expression of pro-inflammatory cytokines is inhibited by the secretion of IL10 (40). These data are interesting, given that patients with MS exhibit reduced levels of IL-10 in mononuclear cells (41). In animal models of MS, it is also possible to observe the anti-inflammatory properties of PBM (27–29). For example, after PBM, a decrease was observed in the levels of pro-inflammatory cytokines, such as interferon and tumor necrosis factor-alpha (TNF-α), and an increase in IL-4 and IL-10 (anti-inflammatory cytokines) (29). Goncalves et al. (28) noted that EAE mice showed profound infiltration of inflammatory cells into the CNS, particularly in the white matter region, and a pronounced increase in IL-17, interferon gamma (IFN-γ), and IL-1b levels. However, PBM reduced the infiltration of inflammatory cells into the CNS of EAE mice. In addition, PBM inhibited the upregulation of IL-17, IFN-γ, and IL-1b. These findings are essential since the secretion of pro-inflammatory cytokines such as IFN-γ and TNF-α initiates and propagates a pro-inflammatory response, generating demyelination of CNS axons by multiple mechanisms, including cytokine-mediated demyelination (42). In this sense, Duarte et al. (27) observed that PBM attenuated the degree of demyelination in the corpus callosum of cuprizone-induced MS model animals, accompanied by a better clinical outcome. In addition, the authors investigated microglial and astrocyte activation. They noted a reduced severity of astrogliosis (GFAP) and microglia (IBA-1) immunoreactivity in the corpus callosum of the cuprizone-induced MS model submitted to PBM treatment. Glial cell activation and inflammatory response are critical hallmarks of MS in humans and animal models (43). About high levels of GFAP, it is known that these high levels in CSF are associated with the progression of MS (44–46) and with clinical disability (44, 46).

### 4.3 Antioxidant and antiapoptotic effects of PBM in MS

Studies show that levels of oxidative stress markers are increased in MS model animals (47–49). However, it is suggested that reduction of oxidative stress prevents the progression of MS, mainly by protecting against apoptosis (50–52). In this sense, in the studies conducted by Muili et al. (30) and Goncalves et al. (28), it was shown that animal models of MS submitted to treatment with PBM exhibit a decrease in oxidative stress markers, such as: nitric oxide (NO), inducible nitric oxide (iNOS) and nitrite. In addition, animal models of MS that received PBM treatment exhibited up-regulation of the Bcl-2 anti-apoptosis gene, an increased Bcl-2: Bax ratio, and reduced apoptosis in the spinal cord (30).

### 4.4 Limitations

Our systematic review presents limitations, mainly because it only analyzed 4 studies with MS patients. The research highlighted in this review shows the therapeutic potential of PBM on MS. However, the lack of details about the PBM parameters used in each work make it difficult to replicate these approaches. Standardization of the PBM protocols would facilitate comparison between the findings of the studies.

## 5 Conclusion

The findings of this systematic review suggest that PBM can be a promising non-pharmacological intervention for MS, as it has been shown to modulate markers linked to inflammation, oxidative stress, and apoptosis. Clinically, PBM has been associated with improvements in motor, sensorial, and cognitive functions in MS patients, indicating its potential as an adjunct therapy to standard MS treatments. No study presented adverse effects of PBM. However, future studies should aim to standardize PBM protocols, assess long-term effects, explore underlying mechanisms, investigate synergistic effects with other treatments, and identify patient subgroups that are most likely to benefit from PBM.

## Data Availability

The original contributions presented in the study are included in the article/supplementary material, further inquiries can be directed to the corresponding author.
